# An improved protocol to establish experimental tuberculous meningitis in the rabbit

**DOI:** 10.1016/j.mex.2020.100832

**Published:** 2020-02-21

**Authors:** Paul O'Brien, Christopher Vinnard, Selvakumar Subbian

**Affiliations:** a Department of Radiation Oncology, Division of Cancer Biology, United States; b Public Health Research Institute of New Jersey Medical School, United States

**Keywords:** Mycobacterium tuberculosis, TB meningitis, Rabbit, Stereotaxic, Infection, Cisterna magna

## Abstract

Tuberculous meningitis (TBM), caused by *Mycobacterium tuberculosis* (Mtb), is the deadliest form of tuberculosis in humans, particularly in children and the geriatric population. However, the host-pathogen interactions underlying TBM is not well understood. Rabbits are a valuable model system to study TB in humans. The rabbit model of TB recapitulates several pathophysiological characteristics, including heterogeneity, architecture, and development of granulomas at the site of infection as observed in Mtb-infected human organs. Previously, our group has established a rabbit model of TBM that has been used to understand the host immune response to Mtb infection and to evaluate novel intervention therapies for TBM. In this model, rabbits infected intracisternally with Mtb showed histopathologic manifestations in the brain and meninges that are hallmarks of TBM in humans, including inflammatory cell accumulation and thickening of the leptomeninges. However, in this model, a helmet made of dental acrylic was attached to rabbit's skull with screws under anesthesia. At 24 h post-procedure, the animals were injected intracisternally with Mtb using a spinal needle. The rabbits were necropsied at various experimental time points up to 2 weeks post-infection. Although this method has been successful in establishing TBM, placement of the dental acrylic helmet on rabbit skull with screws that stays until the experimental endpoint poses stress to the animals and increases the chances of secondary infection. To alleviate these issues, we have developed an improved protocol, in which sedated rabbits are placed on specialised stereotaxic equipment and injected with Mtb intracisternally. This method is less cumbersome, faster, and more efficient in delivering the bacteria. Besides, the animals are not stressed by this method, compared to the previous one.

## Graphical abstract

**Figure:**
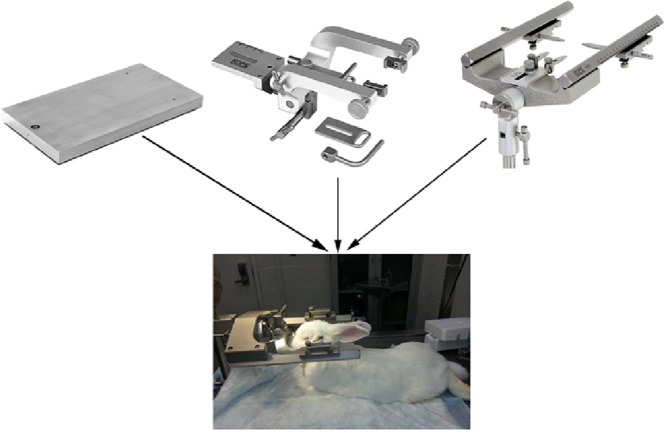

**Table:** Specification Table

Subject Area	*Immunology and Microbiology*
More specific subject area:	*Tuberculosis*
Protocol name:	*Tuberculous meningitis*
Reagents/tools:	*Refer to the main text*
*Experimental design:	*Very brief experimental description*
Trial registration:	*Not applicable*
Ethics:	*Animal procedures described in this study are approved by the institutional biosafety committee and the institutional animal care and use committee of the Rutgers University*
*Value of the Protocol:	*1. Tuberculous meningitis is the deadliest form of tuberculosis with 50% mortality.*
	*2. A rabbit model of TBM recapitulates most of the pathological manifestations seen in patients with TBM.*
	*3. The improved procedure described here to develop TBM in rabbits alleviates the conventional method of placing an acrylic helmet on rabbit skull with screws, which is more stressful.*

## Introduction

Tuberculous meningitis (TBM) constitutes approximately 5%–10% of extra-pulmonary TB cases and about 1% of all active TB cases, but it is the deadliest form of TB as the death rate is alarmingly high (>50%) among all extra-pulmonary TB cases [1,2]. The incidence and prevalence of TBM is higher in children aged below four years and in older people, especially those suffering from severe pulmonary TB and immune-compromised individuals [2], [3]–4]. A recent study shows that about 40% of TBM cases with a deteriorating disease would die within six months of diagnosis [1,5]. Intracerebral inflammation and brain damage are the main characteristics of TBM in humans. The rabbit, as an experimental TBM model of human disease, was established in 1923 [6]. The first TBM rabbit model involved an atlanto-occipital injection of human tubercle bacilli (presumably Mtb), avian tubercle bacilli and bovine tubercle bacilli through the subarachnoid space. The inoculum, prepared in saline solution, did not cause any visible reaction in the rabbits after injection. However, at 6–15 days post-inoculation, tubercles were produced in the meninges accompanied by signs of TBM, such as paralysis, followed by death. This early study demonstrated the resemblance of disease progression and the immunopathology between human TBM and the rabbit model. Later in 1933, a comparative study between children and the rabbit model confirmed the release of Mtb in the meningeal space and affirmed it as the cause for progressive TBM in both systems [7]. Importantly, the clinical and cerebrospinal fluid (CSF) findings in the rabbit TBM model are very similar to human disease, in which lymphocytes are predominant, protein levels are high, and CSF consistency becomes viscous. Moreover, basal meningeal enhancement and hydrocephalus are common abnormalities seen both in the rabbit model of TBM and in the MRI of human TBM cases [8,9].

### Previous procedure to establish human TBM model in rabbits

The severity of disease in TBM was first evaluated by our group using intra-cisternal infection of rabbits with clinical Mtb isolates that are hypervirulent (HN878) or hyper-immunogenic (CDC1551) [9]. In this study, a rabbit model of TBM derived from the original publication by Dacey and Sande was used [10], [11]–12]. In this model, on day-1, the rabbit skull was fit with four aluminum screws of 0.5 × 2 inches under anesthesia with an intramuscular injection of ketamine (0.5 ml/kg) and xylazine (3.5 ml/kg) before bacterial inoculation. These screws were placed over the frontal, parietal and occipital cortex area of the skull; about 1 mm outer to cranial vertex, 2 mm lateral to sagittal suture. A half-turnbuckle helmet was placed on top of the screws using Dura Base dental acrylic. The mask allowed the rabbits to be appropriately positioned in a stereotactic frame. On day-2, the animals were sedated with intramuscular injection of ketamine (0.5 ml/kg) and xylazine (3.5 ml/kg) and using a machine bolt of 0.25 × 4 inches, the head of the animal was placed securely and rigidly in a stereotactic frame that facilitates penetration of the cisterna magna for bacterial inoculation. A spinal needle of 3.5 × 25 G was inserted into the cisterna magna and 0.3 ml of bacterial inoculum, containing about 5 × 10^5^ CFU, was injected. At defined time points post-infection, samples of CSF and blood were collected for downstream analysis [9], [10]–11].

A fundamental limitation of the previous method is the potential for secondary infection caused by the surgical procedure required to attach the acrylic helmet onto the skull. Additionally, animals are sedated multiple times within a short period (~3 days) to place the acrylic helmet and to inoculate the bacteria. Placement of the metallic screw and acrylic helmet and multiple sedations impose stress on the experimental animals. Moreover, the stereotactic equipment used in this model did not have the adapter to hold the rabbit's head in place. This limitation underlies the problematic process of placing the acrylic helmet on the rabbit skull. Here, we describe a new protocol that alleviates the necessity to put the metallic screws and acrylic helmet on the rabbit skull. The critical modification made in the new procedure is the usage of an adapter that fits well with the stereotactic frame and holds the rabbits firmly in place for intracisternal injection. This method also avoids multiple sedation treatment, and the animals would be less stressed.

This procedure is focused on improving the animal welfare by reducing pain and stress. Therefore, we do not anticipate any significant difference in post-Mtb inoculation, such as bacterial CFU, pathology and clinical signs in the brain or CSF between this and old protocol, since both procedures deliver the bacteria in the cisterna magna region. However, we do not have data for method validatation.

## Experimental procedure

### Ethical statement

All animal procedures, including Mtb inoculation were performed in bio-safety level 3 (BSL-3) facilities as per the approved standard operating procedures (SOPs) by the Institutional Animal Care and Use Committee of the Rutgers University. The animals were handled humanely and were fed with food and water ad-libitum and kept in individual cages with 12 h light/dark cycle.

Here we describe a step-wise procedure for an improved rabbit model of TBM

**Fig. 1:**
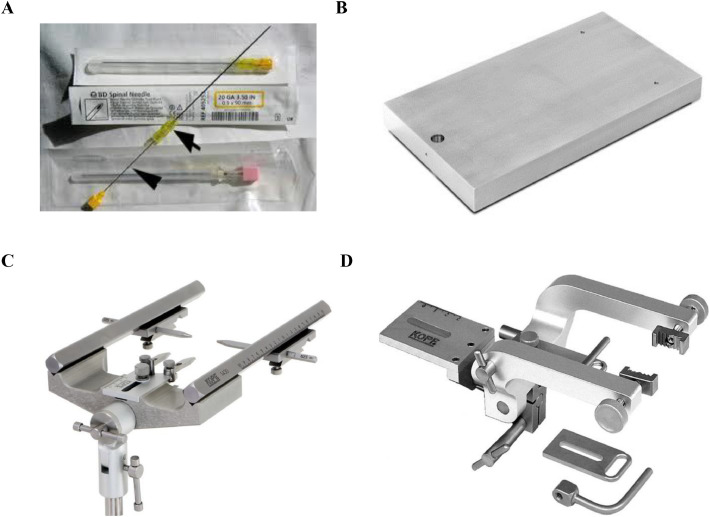
Images of the spinal needle (**A**) with sheath (arrow) and central guide-wire (arrow-head) used for bacterial inoculation; Baseplate (B), holding unit (**C**) and adaptor (D) of the rabbit stereotaxic instrument. While B, C and D are components of the stereotaxic instrument, A is the needle used to inoculate bacteria into the cisterna magna area of the rabbit brain. Images B–D are the courtesy of Kopf Instruments, CA, USA.
  8. The rabbit should be secured in the stereotaxic frame, on the downdraft table, to facilitate the intracisternal tap (Fig. 2). The dorsal aspect of the rabbit neck is palpated to locate the correct position of the cisterna magna (a depressed area of about 5 mm in diameter, inferior to the occipital protuberance). The area of injection is then cleaned with 70% ethanol.

A. *Materials:*
  Class II A biological safety cabinet and Class II B biological safety cabinet
  Incubators (Humidified and at 37 °C)
  Filter-isolated rat cages
  Trays/containers for specimen transport
  Moveable carts
  Stereotaxic Frame
  25 G Spinal Needles
  Weighing scale
  Probe sonicator
  Water bath sonicator
  Vortex
  Disposable Tyvek Suit
  Tyvek Aprons
  Tyvek Aprons with Sleeves
  Tyvek Sleeves
  Booties
  Gloves, Nitrile
  Breathing Tube PAPR
  Powered air-purifying respirators (PAPR) with a full skirt
  Glycerol
  Tween 80
  OADC enrichment
  Sterile saline
  Distilled Water
  1X PBS with Tween
  1X PBS
  70% Ethanol
  10% Clorox, freshly prepared
  10% Formalin
  Vesphene
  Decon Wipes
  Blue Absorbent Pads
  Formalin Spill Pads
  Large Autoclave Bags
  Autoclave Tape
  White Boxes
  15 mL and 50 mL Conical tubes
  Plastic containers for tissue samples
  200uL and 1000 mL Pipettes
  P200 aerosol barrier tips
  P1000 aerosol barrier tips
  Middlebrook 7H11 agar
  Petri plates, disposable 15 × 100 mm
  Polyethylene bags
  *M. tuberculosis* HN878 or CDC1551 strains
  BD 23G3/4 Needles
  BD 18 G 1 1/2 Needles
  Heparin
  BD Syringe 1 mL
  BD Syringe 3 mL
  BD Syringe 20 mL
  Point-lock Needle Protection Device
  10 mL EDTA Tube (Purple Top)
  10 mL Heparin Tube (Green Top)
  Single-use scalpels blade size No 21.
  Small Sharps Container
  Large Sharps Container
  Electrical shaver / clipper with size #40 blade
  Surgical instrument (forceps, scissors, shear cutters)
  1.8 mL cryovials
  Dry ice
  Regular ice
  *Note:*
  All of the following procedures should be performed in Class II Biosafety Cabinets (BSC-2). Rabbits are housed in individual negative pressure cages, and the cages are placed in negative pressure rooms. Before performing the infection experiment in animals, a sign must be hung on the door of the biosafety module alerting other persons not to enter during the procedure.
  Personal Protective Equipment: disposable Tyvek suit, hair-bonnet; eye protection, disposable booties, and double gloves. Powered Air Purifying Respirator (PAPR) with a full skirt should be used for: (a) infecting the animals; (b) working with the infectious suspension, and (c) necropsy. A regular N95 mask may be used for the rest of the procedures.
B. *Preparing the animal for infection:*
  1. Specific-pathogen free, New Zealand White rabbits of 2.4–2.6 kg body weight are individually housed for about a week for acclimatisation in the BSL2/BSL3 animal facility.
  2. On the day of infection, rabbits are moved to BSL3 facility and sedated with Ketamine (35 mg/kg) plus Xylazine (5 mg/kg) by intramuscular injection. Once sedated, the animals are removed to the procedure room by means of a filter-isolated crate.
  3. The fur around the occipital region of the rabbit's head is shaved using an electrical clipper.
  4. The animal is placed in a stereotaxic frame, on a stainless steel table, to facilitate the intracisternal tap.
C. *Preparation of bacterial inoculum:*
  5. A suspension of Mycobacterium tuberculosis at a density of about 5 × 10^6^ CFU/ml in sterile saline of 1x PBS should be prepared in advance.
  6. The bacterial suspension is then aspirated into a 1 cc syringe, inside the biosafety cabinet. The needle is disconnected, using a special safety device (Point-lock Needle Protection Devise, Fisher # 22-004-139), placed next to the animal frame.
  7. The central guide-wire (stylet) of a 25 G spinal needle (Fig. 1A) is removed, and the plunger of the inoculum-containing 1 cc syringe is pulled back to allow about 0.2 cc air barrier between the syringe tip and the inoculum. The needle is carefully detached from the 1 cc syringe and is correctly attached to the 25 G spinal needle with the protective sheath remaining on the needle. The syringe with spinal needle and bacterial inoculum is placed inside a lidded, leak-proof transfer container on top of a paper towel soaked with disinfectant. The container is sealed and wiped on the outside with DeconPlus Wipes then removed from the BSC and placed next to the stereotaxic frame on the downdraft table (Fig. 1B–D).

**Fig. 2:**
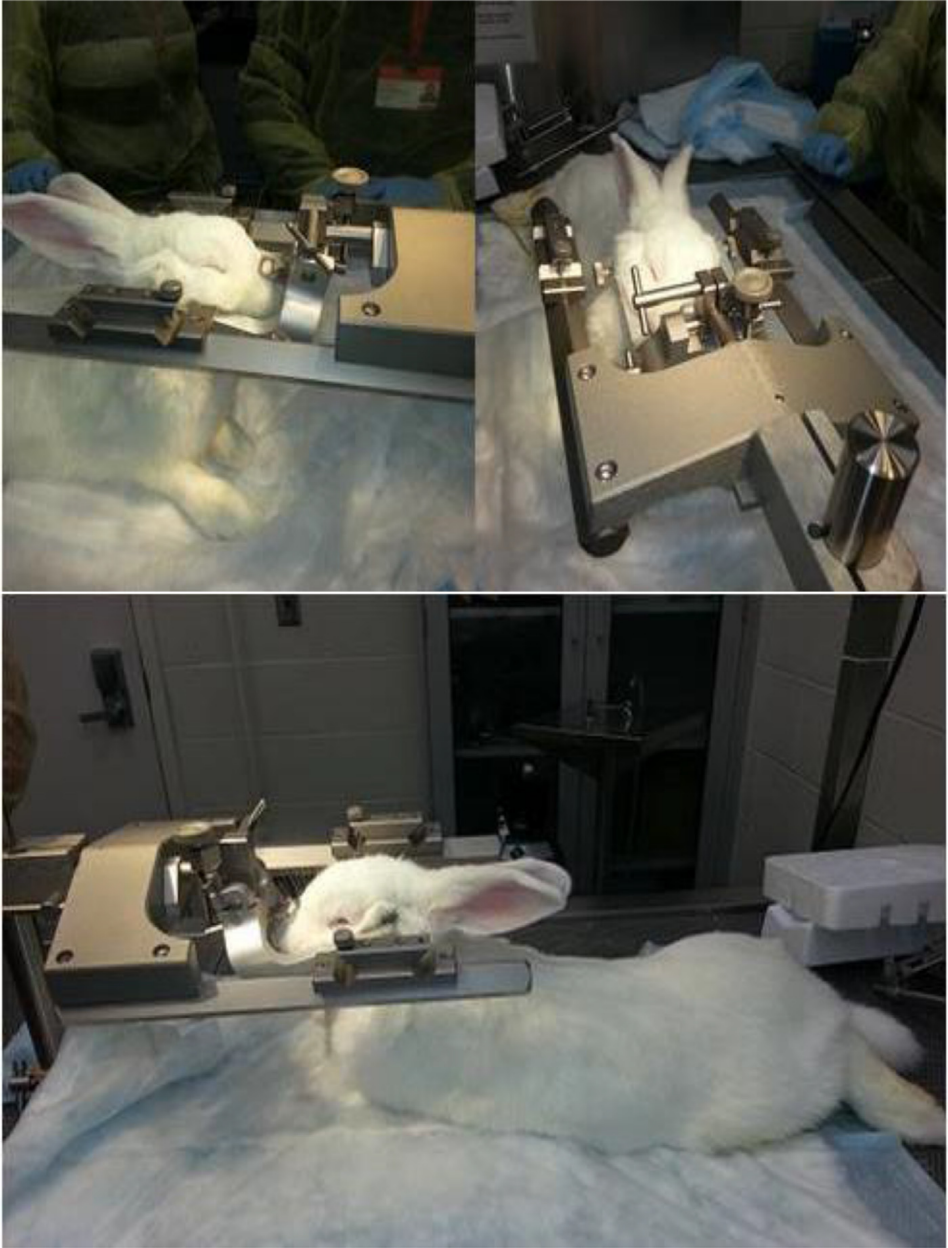
Images showing the placement of a sedated rabbit in the stereotaxic instrument.
  9. A sample of CSF (0.3 ml) is withdrawn using a sterile spinal needle fit on a 1cc syringe. This procedure is done as follows: the protected sheath of the spinal needle is removed and holding the syringe at the base, and the needle is gradually advanced horizontally in 5 mm increments at the previously identified position. The needle pushes through the soft tissue until the appropriate loss of resistance is felt. The needle position within the cisterna magna is ensured when the syringe plunger is slowly withdrawn; the absence of blood and flow of CSF confirms the correct positioning (0.05 mL is sufficient to visualise within the syringe).
  10. The syringe and needle are removed, and the CSF sample is placed in a 1.5 ml microtube inside the biosafety cabinet. The used syringe is discarded in the sharp container. 20µl of the sample is added to a special cuvette with a lid, filled with buffer, to measure the number of WBCs, using a Coulter cell counter.
D. *Inoculation of bacteria:*
  11. The spinal needle fit on 1cc syringe containing 0.2 ml bacterial inoculum suspension prepared in step 7, is removed from the transfer container, and the plunger is carefully pushed in to remove any air at the tip. A saturated towel or wipe with a tuberculocidal disinfectant is used to wipe the syringe if a droplet is formed on the syringe tip.
  12. The needle is gradually advanced horizontally, holding the syringe at the base, at 0.5 cm increments at the previously identified position as in step 9. Following this, the bacterial inoculum is slowly injected over 20–30 s (Fig. 3).

**Fig. 3:**
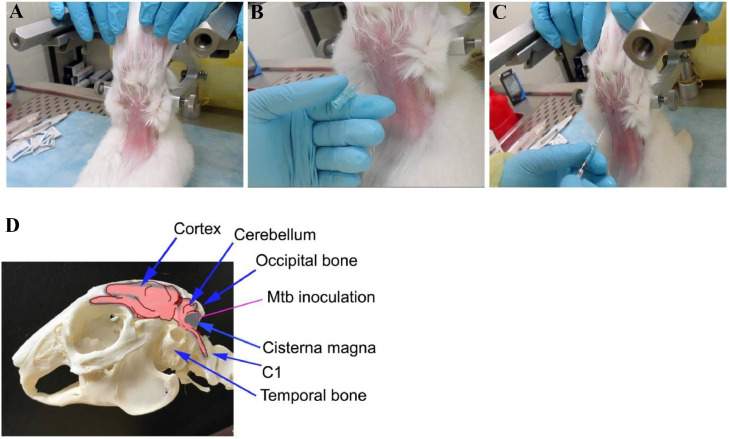
Images showing the position for intracisternal injection (A), needle placement (B) and delivery of bacteria (C) in a sedated rabbit immobilised on a stereotaxic instrument. (D) is the diagrammatic view of the rabbit skull with brain parts, including the cisterna magna (Mtb inoculation site).
  13. The empty syringe with attached spinal needle is carefully removed and placed immediately into the sharps bin.
  14. A block of Gauze or paper towel may be applied to the injection site with slight pressure to stop any bleeding if required. The gauze or paper towel is discarded into the biohazard waste bag.
  15. The rabbit is removed from the stereotaxic unit and returned to the housing room in filter-isolated individual crates, and placed in the cages. At this point, they are still under anesthesia.
  16. Once all the animals are inoculated with Mtb, the stereotaxic frame, down-draft table, and carts are appropriately disinfected with 10% Clorox Bleach allowing for 20 min of contact time before rinsing the stereotaxic structure, down-draft table with water, and wiping the carts with 70% ethanol.
